# Synaptic plasticity in depression: from mice to humans

**DOI:** 10.1192/j.eurpsy.2023.71

**Published:** 2023-07-19

**Authors:** S. Vestring

**Affiliations:** Department of Psychiatry and Psychotherapy, University Clinic Freiburg, Freiburg, Germany

## Abstract

**Abstract:**

In the last decade, neuroplasticity has become largely accepted in the etiology and treatment of mood disorders. Animal models of depression showed that severe stress downregulates many forms of plasticity, resulting in an inhibition of long-term potentiation (LTP), a facilitation of long-term depression (LTD) and an impairment of synaptic transmission. Essentially all treatments for mood disorders, including the rapid acting antidepressant ketamine, promote neuroplasticity and plasticity plays a critical mechanistic role in recovery. Therefore, a targeted intervention of LTP/LTD pathways by small molecules or highly specific RNA therapeutics could lead the way to novel and fast acting antidepressants. For instance, an RNA-based modulation of N-methyl-d-aspartate receptor subunits rescued LTP and exerted rapid antidepressive effects in mice models of depression. The translation of such principal, the rescue of plasticity as an antidepressive intervention, from rodents to humans is an ongoing challenge. However, various indirect assessment methods of plasticity in humans, like visually evoked (VEP) potentials and transcranial magnetic stimulation (TMS)-based paired associative stimulation paradigms revealed an impairment of plasticity in depressed humans, which was found corrected after effective treatment.

**Disclosure of Interest:**

None Declared

